# Budd-Chiari syndrome following abdominal trauma

**DOI:** 10.21542/gcsp.2024.19

**Published:** 2024-04-20

**Authors:** Feridoun Sabzi, Reza Faraji

**Affiliations:** 1 Department of General Surgery, School of Medicine, Kermanshah University of Medical Sciences, Kermanshah, Iran; 2 Tuberculosis and Lung Diseases Research Center, Ilam University of Medical Sciences, Ilam, Iran

## Abstract

We report a case of Budd-Chiari syndrome (BCS) in a 44-year-old man with inferior vena cava (IVC) thrombosis and nephrotic syndrome. This case was complicated by right atrial clot and pulmonary emboli. Endothelial injury of the IVC was the likely mechanism, following a kick from a donkey. Abdominal ultrasonography revealed a large thrombosis located in a segment of IVC near its orifice in the right atrium. Transthoracic echocardiography (TTE) revealed IVC thrombosis that extended to the right atrium; however, pulmonary emboli (PE) were not documented in TTE. Intraoperative exploration revealed multiple clots in the main and left pulmonary artery branches. The patient recovered well after open-heart surgery with resection of the right atrium, IVC, and pulmonary artery emboli. BCS should be routinely considered for patients with nephrotic syndrome.

## Introduction

The primary cause of IVC thrombosis is typically the formation of an embolus originating from deep vein thrombosis (DVT) in the extremities. This embolus can then travel to the IVC and cause blockage or thrombosis.

IVC thrombosis resulting from blunt abdominal trauma (from a donkey kick) is not only an interesting case but also a rare phenomenon. However, in many subjects, post-traumatic IVC thrombosis is not clinically diagnosed by cardiologists^1^. Generally, this complication could be a dilemma for internists, who are first-line clinicians. The true incidence of post-traumatic IVC thrombosis is unknown, but seems to be between 0.5 and 1 percent in all patients with multiple traumas who have stayed in the hospital.

This complication is more common in elderly patients, and in women it can be attributed to the weakening of the abdominal wall due to atrophy of the abdominal muscles^2^.

Other common predictors of IVC thrombosis identified in hospitalized individuals, which may occur alongside nephrotic syndrome, include certain malignancies. These malignancies may occasionally coexist with abdominal trauma.

### Case presentation

A 42-year-old male shepherd presented to our hospital with a one-week history of blunt abdominal trauma following a kick from a donkey, which was followed by sciatic-like loin pain, lower back bruising, pitting edema of both lower extremities, dyspnea, and fever of 39 °C.

On physical examination, his blood pressure and pulse rate were 120/70, 120 respectively, and the abdomen examination revealed a soft but not tender abdomen, but mild tenderness was found in the right upper quadrant of the right lobe of the liver.

The chest examination was normal without crackles or rales. With the exception of mild bruising of the anterior abdominal wall, no hematoma was detected on laboratory examination. This also revealed no anemia and normal serum lactate dehydrogenase levels. However, C-reactive protein level had increased to 220 mg/L. Other coagulation and thrombophilia examinations with concomitant biochemical tests were unremarkable.

Lower limb arterial pulsations were normal. Chest radiography, and the abdomen and lumbar spine were normal. Laboratory examination revealed unremarkable concentrations of urea (20 mg/dl), creatinine (1.5 mg/dl), sodium (130 mmol/L), and potassium (3.6 mmol/L).

Urine exam with a urine dipstick showed positivity of protein, which equated to ≥3 g urinary protein daily. Serum albumin level was also reduced due to hypoalbuminemia on serum examination. Doppler ultrasound examination of both femoral veins revealed marked expansion of both veins; however, an intraluminal thrombus was not detected.

**Figure 1.**
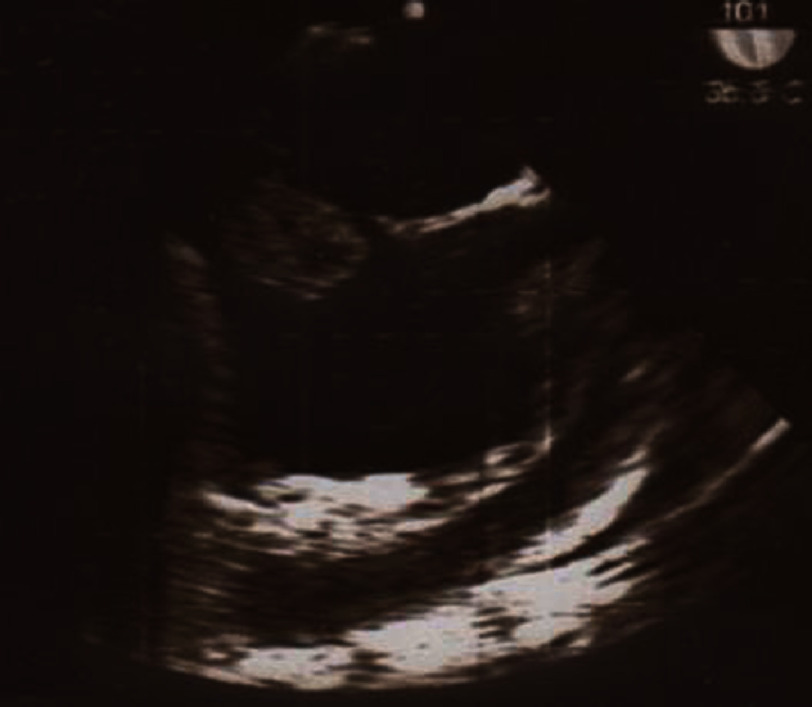
Transesophageal echocardiography showing thrombosis in opening of inferior vena cava.

**Figure 2.**
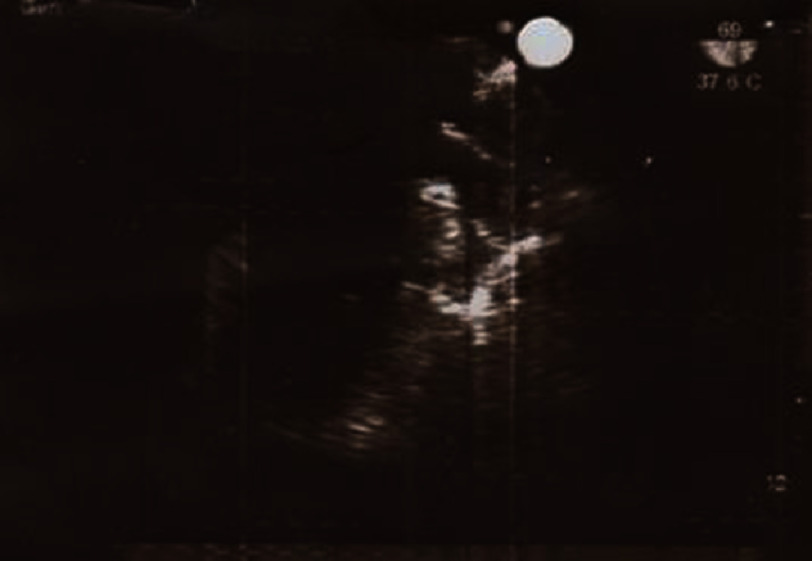
Transesophageal echocardiography from a different angle showing thrombosis in opening of inferior vena cava.

Transthoracic echocardiography revealed a free-floating clot-like mass in the IVC opening to the RA extending to the wall of the RA (Figures 1 and 2). Despite a poor view on abdominal ultrasound, a possible clot in the suprahepatic segment of the IVC was detected.

Other segments of the IVC and iliac veins were engorged. No mass was found in the hepatic or biliary system, or in the retroperitoneal space. The patient was scheduled for emergency removal of the clot by open-heart surgery to prevent thrombotic emboli. A median sternotomy was performed, and access to the heart was obtained *via* vertical pericardiotomy.

Cardiopulmonary bypass (CPB) was instituted with aortic and bicaval cannulation. The pulmonary artery was then released and snared with tape. With severe hypothermia, cardioplegic arrest was observed along with cold cardioplegia. With the establishment of total circulatory arrest, the right atrium (RA) was incised through the atrioventricular groove and the clot was removed.

The IVC opening was explored, and no ring, stenosis band, or congenital web was found. The IVC was thoroughly irrigated with copious amounts of normal saline (NS). CPB was temporarily re-established to flush out any remaining clots in the IVC. The lower IVC cannula was introduced into the IVC, and the IVC was snared. The main pulmonary artery was explored and both pulmonary artery branches were suctioned. During irrigation, a clot ( 1  × 2 cm) was found at the tip of the suction tube in the right pulmonary artery. Circulation was fully established and the RA was closed.

**Figure 3.**
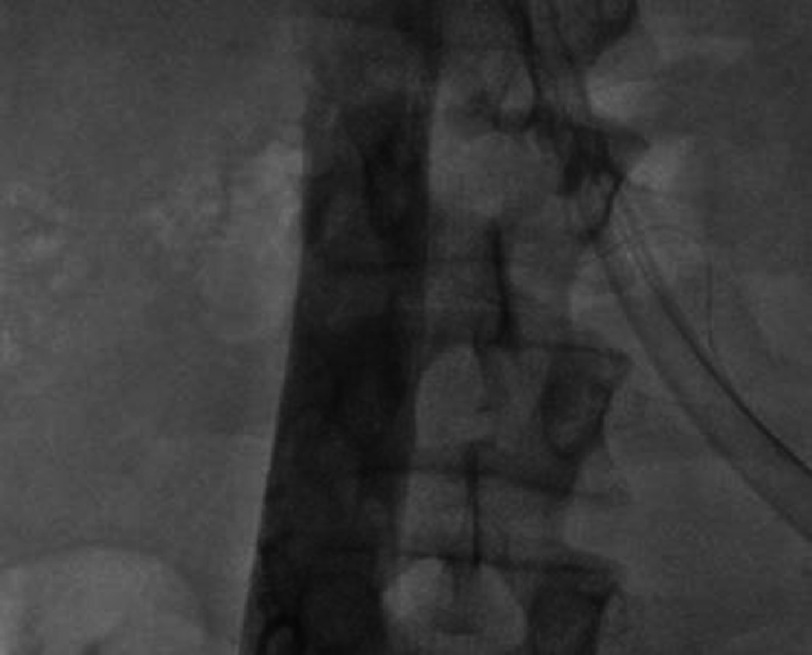
Showing patency of IVC in angiography.

**Figure 4.**
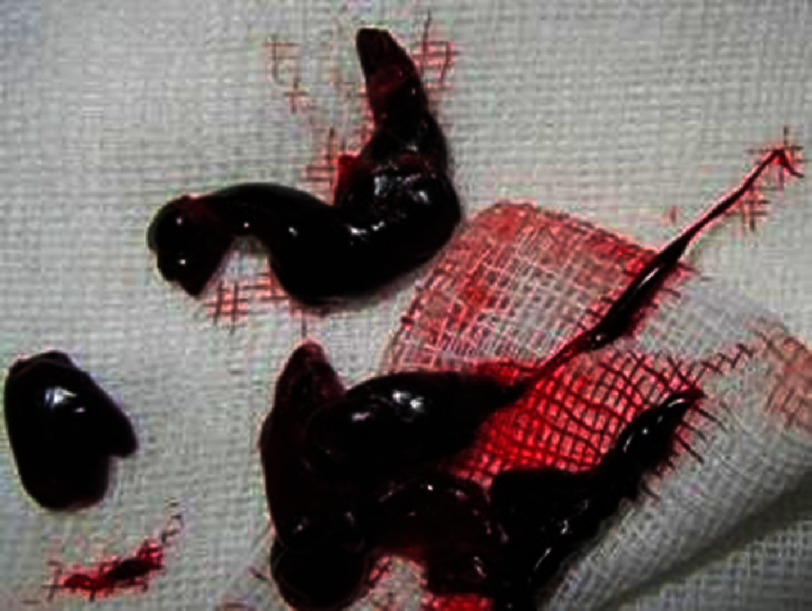
Clots removed in gross pathology.

Peri- and post-operative conditions were unremarkable. The total circulatory arrest time was 9 min. Lower extremity edema and the presence of nephrotic syndrome prompted us to recommend an IVC venogram in the postoperative period to identify patency of the lower segment of the IVC renal and hepatic veins (Figures 3 and 4). Anticoagulation with oral warfarin was initiated in the morning following surgery and continued for 4 weeks. At the seventh month of follow-up, physical examination was normal, and there was no evidence of recurrent thrombosis RA or proteinuria on TTE and urine analysis. His lower extremity sciatic-like pain and edema disappeared some days after surgery, and the patient remained in good condition at the ninth month of follow-up.

## Discussion

IVC clotting is commonly associated with malignancy. Stein^3^ showed that carcinomas were found in 40% of subjects with radiological diagnosis of IVC clotting. The most common factor in the occurrence of IVC thrombosis is renal carcinoma, followed by conditions affecting the lungs and trachea.

One of the important causes of IVC thrombosis that may be missed with concomitant trauma is the formation of a congenital web in the IVC that presents as nephrotic syndrome with concomitant hypercoagulability states, such as trauma^4,5^.

Most stenotic congenital rings or webs in the IVC are asymptomatic because of the presence of extensive collateral veins from the IVC to the neighboring veins. However, in traumatic IVC thrombosis, endothelial injury, and thrombosis in the lower part of the IVC, the origin of the collateral veins blocks the flow of the retrograde stream to the upper segment, leading to Budd-Chiari syndrome.

Acquired injury of endothelium in the IVC in cases of blunt abdominal trauma should be accompanied by spontaneous clot formation in an untraumatized IVC segment. In some cases, clotting of the IVC is caused by an external mass that compresses the IVC lumen and constricts its lumen, or clot formation due to pathologic endothelial injury within the lumen wall^6^.

Pathological overlap could be seen between the congenital stenosis web and acquired injury, and between different components of Virchow’s triad. Spontaneous IVC thrombosis is associated with chronic debility and has been reported in elderly subjects with abnormal IVC due to a state of thrombophilia underlying the pathological process^7,8^.

Spontaneous IVC clotting in malignancy of the renal system is related to the prothrombotic state induced by the tumor. These thrombophilia states may be accompanied by subsequent thrombotic states of chemotherapy- or radiotherapy-induced endothelial injury, which enhances the risk of IVC clotting.

A rare primary IVC carcinoma and neighboring carcinoma-like renal carcinomas can also invade the IVC wall *via* renal vein infiltration, causing endothelial injury and subsequent clotting^9–12^. Intraluminal tumor infiltration may extend upward or downward intraluminally in the direction of the renal vein into the IVC and could extend as a long segment mass as far as the entrance of the heart or right atrium, resulting in thrombosis in the RA or emboli to the pulmonary system.

Abdominal trauma, as in our case, may cause endothelial IVC injury secondary to direct shearing trauma, compression of the hematoma in neighboring organs, or, rarely, penetrating injury.

Indirect trauma has been documented to be caused by falls from a height, crush injuries from an earthquake, car accidents by steering wheel impact, boxing and kicking to the abdomen, weight lifting, athletic competitions, anabolic steroid abuse in body builders, blunt spinal trauma leading to IVC endothelial injury, or bruising from spinal trauma^13–16^.

Minor injuries acquired during seemingly innocent tasks, such as replacing a car tire, tumbling, and cycling, can compress the IVC and lead to IVC thrombosis^17,18^.

Compressive narrowing of the IVC causes turbulence of IVC blood flow and venous stasis below the stenotic segment, which facilitates the occurrence of clot^6^. Such IVC clotting can be triggered by large lymph nodes in lymphoma, idiopathic retroperitoneal fibrosis, distortion of the IVC, ruptured aortic aneurysms or dissection, and huge abdominal masses.

Other common causes of DVT that may predispose to IVC clotting include congenital or acquired thrombophilic states (SLE), as well as secondary variables such as oral contraceptives, hormonal changes during pregnancy, high body index, carcinoma, and chronic inflammatory states^19–22^. Crohn’s disease, ulcerative colitis, primary nephrotic syndrome, and variable infectious diseases predispose to IVC thromboses.

There are also associated risk factors such as dehydration, protein-losing enteropathy, thrombophilia, and increased inflammatory reactions in response to chronic sepsis^23^. A rare type of IVC clotting, known as obliterative hepatocavopathy, has been observed in the hepatic segment of the IVC and is defined by an organizing thrombus leading to the formation of a porous membrane that limits the flow of the IVC. This pathology is endemic in a suburb of Nepal, where some authors have diagnosed infection by *Staphylococcus aureus* as a cause of thrombophlebitis in the IVC and consequent fibrotic stenosis^24^.

### What have we learnt?

Budd-Chiari syndrome with inferior vena cava thrombosis and nephrotic syndrome is very complicated. In this case, it was complicated by a right atrial clot and pulmonary emboli. Endothelial injury to the IVC is a likely mechanism. The presence of a clot at the entrance to the right atrium, accompanied by lower back pain and sciatic symptoms in the lower extremities, with a history of blunt trauma to the abdomen, may be a rare presentation of BCS.

### Funding

This research has not received any specific grant from public, commercial, or non-profit sector agencies.

## Conflicts of interest

The authors have no conflicts of interest to declare.
